# The Principle of Nanomaterials Based Surface Plasmon Resonance Biosensors and Its Potential for Dopamine Detection

**DOI:** 10.3390/molecules25122769

**Published:** 2020-06-15

**Authors:** Faten Bashar Kamal Eddin, Yap Wing Fen

**Affiliations:** 1 Department of Physics, Faculty of Science, University Putra Malaysia, UPM, Serdang 43400, Selangor, Malaysia; faten.mphy@gmail.com; 2 Functional Devices Laboratory, Institute of Advanced Technology, University Putra Malaysia, UPM, Serdang 43400, Selangor, Malaysia

**Keywords:** neurotransmitters, nanomaterials, surface plasmon resonance, optical, biosensors, diagnosis, dopamine

## Abstract

For a healthy life, the human biological system should work in order. Scheduled lifestyle and lack of nutrients usually lead to fluctuations in the biological entities levels such as neurotransmitters (NTs), proteins, and hormones, which in turns put the human health in risk. Dopamine (DA) is an extremely important catecholamine NT distributed in the central nervous system. Its level in the body controls the function of human metabolism, central nervous, renal, hormonal, and cardiovascular systems. It is closely related to the major domains of human cognition, feeling, and human desires, as well as learning. Several neurological disorders such as schizophrenia and Parkinson’s disease are related to the extreme abnormalities in DA levels. Therefore, the development of an accurate, effective, and highly sensitive method for rapid determination of DA concentrations is desired. Up to now, different methods have been reported for DA detection such as electrochemical strategies, high-performance liquid chromatography, colorimetry, and capillary electrophoresis mass spectrometry. However, most of them have some limitations. Surface plasmon resonance (SPR) spectroscopy was widely used in biosensing. However, its use to detect NTs is still growing and has fascinated impressive attention of the scientific community. The focus in this concise review paper will be on the principle of SPR sensors and its operation mechanism, the factors that affect the sensor performance. The efficiency of SPR biosensors to detect several clinically related analytes will be mentioned. DA functions in the human body will be explained. Additionally, this review will cover the incorporation of nanomaterials into SPR biosensors and its potential for DA sensing with mention to its advantages and disadvantages.

## 1. Introduction

Over the last few decades there has been a great effort towards the development of label-free optical biosensors. These essential analytical tools offer real-time analysis, detection of chemical and biological species with high sensitivity and selectivity. The tremendous advances in these biosensors will have a major impact on our health care. Among these technologies used to analyze the bio-specific interactions, surface plasmon resonance (SPR) biosensors today belong to the most advanced [1]. It has proven effective in medical diagnostics [2,3], food quality tests [4], detection of heavy metal ions [5], and others with respect to environmental protection.

Comparing to the conventional diagnostic tools, SPR biosensors have multiple advantages such as easy preparation, no requirement of labeling, real-time detection capability, cost- effectiveness, and high specificity and sensitivity. However, for the label-free detection of low concentrations of analytes with small molecular weight its sensitivity is not enough. Therefore, considerable efforts have been invested to overcome these challenges and improve the sensitivity of the SPR biosensor with keeping all its advantages. Nanomaterials are promising candidates and have demonstrated their appropriateness in the biosensing field. All nanomaterials have a general feature, which is the high specific surface. This enables the immobilization of an enhanced amount of bioreceptor units. Using the functional nanomaterials significantly enhanced the sensors performances, increased the sensitivity and selectivity of the sensing platform. The sensing performance is affected by synthetic procedure of the used nanomaterial, its shape and size. Additionally, the immobilization strategy used to functionalize the sensor chip is still challenge [6]. The purpose of this concise review is to introduce SPR concepts, and simplify the mechanism of SPR based sensor from dip to real-time measurements, explain the important characteristics in SPR sensor performance, mention several clinically related analytes that have been detected using SPR biosensors efficiently. Additionally, this mini review will explain the critical role of dopamine (DA) in human body and the potential of nanomaterials based SPR biosensors to detect it with mentioning its advantages and disadvantages.

## 2. SPR Phenomena

**Figure 1:**
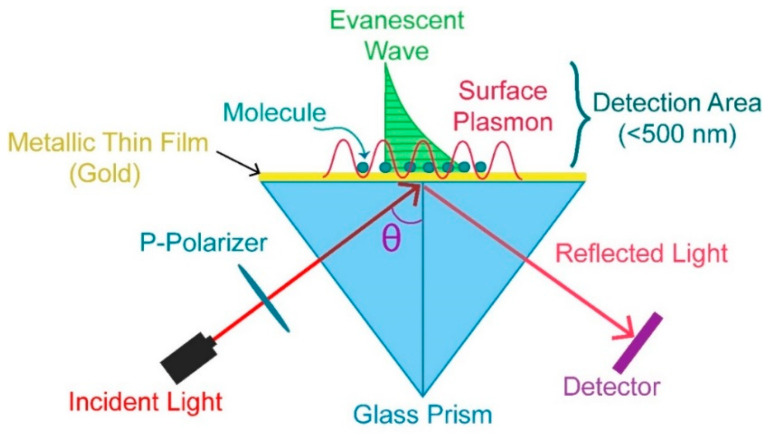
Experimental set-up of surface plasmons (SPs) excitation.

SPR is a quantum electromagnetic phenomenon that occurs when light interacts with free electrons at the interface between the metal and dielectric [7,8]. This optical process happens when monochromatic and p-polarized light beam strikes the surface of metal (typically gold) as shown in Figure 1.

**Figure 2:**
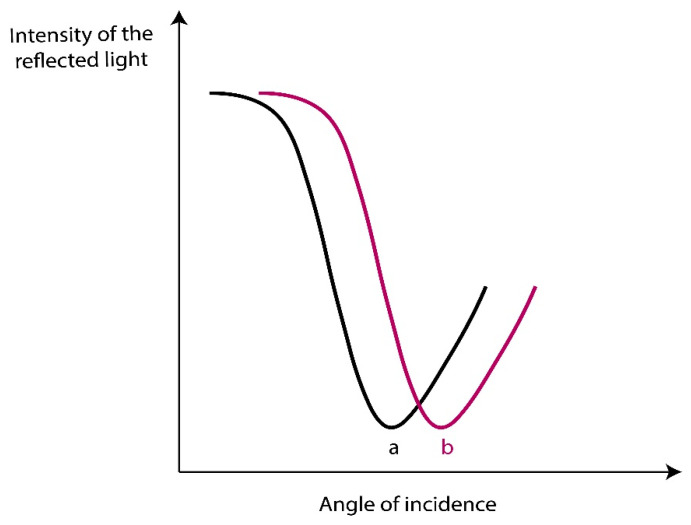
(**a**) A dip in the intensity of the reflected light after SPs excitation and (**b**) an angular shift from a to b due to a refractive index (RI) change on the Au film.

At a specific incidence angle when light satisfies resonance conditions and the frequency of the incident light matches the frequency of the surface plasmon wave, the light energy partially transfers to the electron packages on the metallic surface. After that, the observed reflected light shows a dip in the intensity as shown in Figure 2a. The electron coherent oscillations that were excited by exponentially decaying evanescent field of the incident light are called surface plasmons (SPs) and propagate parallel to the metallic surface. The angle at which the reflected light shows the maximum loss of intensity is called resonance angle or SPR-dip [9,10]. 

At the beginning of the twentieth century in 1902, the first observation of SPs was obtained by Wood, who reported anomalies in the diffraction spectrum of polychromatic light on a metallic diffraction grating [11]. Then, the connection between these abnormal diffractions and the excitation of electromagnetic surface waves on the diffraction grating surface was proved by Fano [12]. The clear explanation of this phenomenon was not complete until 1968, when Otto verified the concept experimentally and proved that in the attenuated total reflection (ATR) method, the excitation of SPs led to drop in the reflectivity [13]. Before the end of the same year, Kretschmann and Raether made some modifications on the configuration of ATR method to observe the excitation of SPs [14]. These important achievements done by Otto, Kretschmann, and Raether established an appropriate method to excite SPs and ushered in a promising future in modern optics. There are two categories of SPs, propagating SPs (PSPs), and localized SPs (LSPs). The excitation of SPs in the first category occurs on the metallic films. There are several approaches for this type of SPs such as the Kretschmann and Otto prism coupler, optical waveguides coupler, diffraction gratings, and optical fiber coupler. While the excitation of LSPs occurs on metallic nanoparticles [15]. The most common approach used in triggering SPs is prism coupling, which is also known as the ATR mode [16]. In Otto configuration, there is a certain distance separates the prism and the thin metallic film, the refractive index (RI) of this dielectric layer is small. The Kretschmann configuration is the easiest one. In this geometry, the prism is in contact with the metallic layer directly, and the wave vector component of SPs propagating along the interface is coupled with the wave vector of the incident photon. The practical performance of the sensor is confined to the resonance angle. The sensitivity of SPR sensors based on prisms is higher than that of SPR sensors based on grating couplers. Additionally, prism-based SPR sensors using wavelength interrogation provide the best resolution and a great deal of flexibility in terms of the analyte refractive index (RI) covered [17]. So, it has become a highly efficient mechanism for optical sensing of several biological, chemical and environmental changes.

## 3. SPR Based Sensor

SPR is an excellent method to monitor changes occurring in the RI in the near vicinity of the metal surface. When the RI changes as a result of adsorption of molecules on the metal surface, the resonance spectral response of the SPR will change, and thus shifting angular or spectral position of the SPR dip will happen as shown in Figure 2b to reflect certain properties of the system and provide information on the kinetics of molecules adsorbed on the surface.

**Figure 3:**
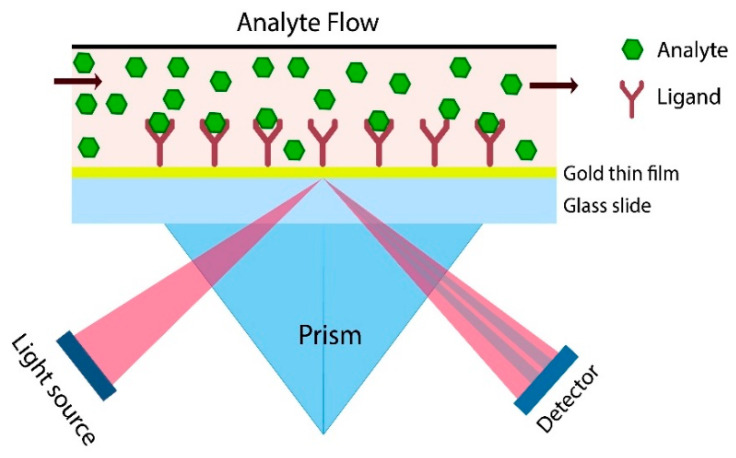
Direct label free detection.

SPR sensors lack intrinsic selectivity, the change of the signal depends on all RI changes in the evanescent field. Changing the buffer composition or concentration leads to RI difference of the medium. Additionally, this depends on the medium temperature and the non-specific and specific adsorption of molecules on the SPR chips. The enhancement of the SPR biosensor needs modification of its surface with suitable ligands to capture the target compound (the analyte) and neglect other molecules available in the sample as shown in Figure 3. These ligands can be permanently or temporarily immobilized on the sensor surface. The analyte accumulation results in a RI change in the evanescent field detected. When the ligand captures the analyte, the measurable signal rises and this is called direct label free detection.

**Figure 4:**
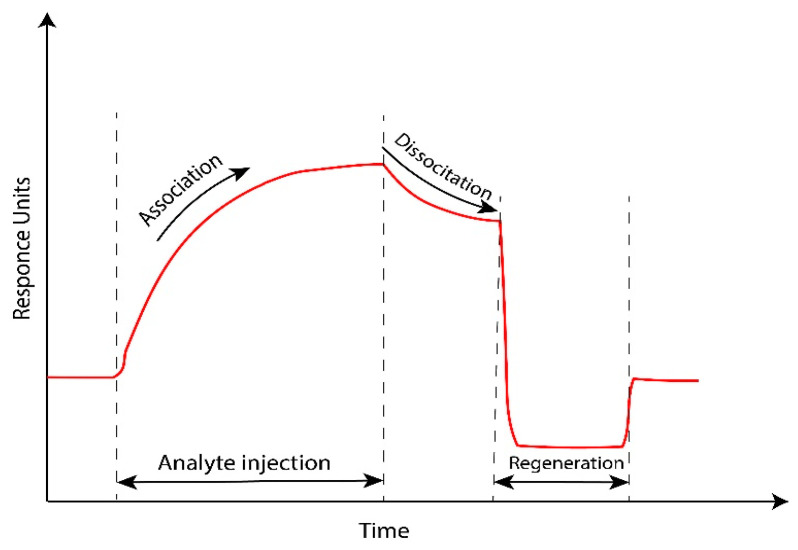
Surface plasmon resonance (SPR) sensogram.

Following in time the resonance angle or wave length shift at which the dip is observed produces the sensogram (Figure 4), then the amount of adsorbed species after injection of the original baseline buffer can be determined, and a study of the kinetics of the biomolecular interaction can be done. The sensor surface should be conditioned with a suitable buffer solution to start each measurement. It is essential to have a stable base line first [18]. After analyte injection, the association phase starts and the ligands on the surface of the sensor capture the target compounds. Upon injection of the baseline, the dissociation of target compounds and non-specifically binding molecules from the surface start. To break the specific binding between the analyte and ligands, regeneration solution should be injected. This step is vital in order to perform many tests using the same sensor surface. 

## 4. The Important Characteristics in SPR Sensor Performance

To evaluate the performance of the SPR sensor, there are several major characteristics that should be taken in consideration. The main performance indicators include the sensor sensitivity, resolution, accuracy, reproducibility, repeatability, and limit of detection (LOD) [19]. The ratio of the change in SPR sensor output (e.g., angle of incidence, wavelength, amplitude) to the change in the measurand (e.g., RI, analyte concentration, and thickness) represents the sensitivity of the sensor. The smallest change in the RI that produces a detectable change in the SPR sensor output defines the sensor resolution. The accuracy defines the degree to which the sensor readout value corresponds to the actual value of the measurand. Sensor’s precision also includes reproducibility, which shows whether entire measurements can be reproduced in its entirety. Additionally, the repeatability is a way to measure the sensor precision. It shows the sensor ability to reproduce the same response under the same conditions over many repetitions. The LOD means the lowest concentration of analyte that can be detected by the sensor.

The shape of the SPR response curve affects the two important performance parameters, sensitivity and signal-to-noise ratio (SNR). Sharp SPR-dip is desirable because the narrower the width, the higher the detection accuracy and the deeper the curve, the higher the sensitivity. The sensor design parameters such as light polarization modes, light coupling techniques, types of metals influence the width and depth of the SPR dip. In prism-based SPR sensor, the physical structure of prism used (triangular, conical, hemispherical, and half cylindrical) and its RI affects the SPR curve [20]. Among the parameters that play a prominent role in the development of a highly sensitive SPR sensor, the thickness of the metallic thin films must be mentioned [21]. Until now, the ideal thickness of prepared layers to generate maximum SPR is within the range of 40 nm to 60 nm [22,23]. Additionally, the used wavelengths for excitation strongly affect the resonance curve width. Narrowing the reflectance curve necessarily increases the SPR propagation length [24,25]. Using the excitation wavelength in the IR region results in an increase in the penetration depth with the consequence that the reflectance minimum will become more sensitive to dielectric changes relatively far from the metal/dielectric interface; thus, the SPR signal gets weaker and the surface-sensitive character of SPR becomes less prominent. The usage of red laser, λ = 633 nm significantly enhances the maximum excitation of SPs and leads to strong SPR signal, while the excitation wavelength in UV region generates a very weak SPR signal [26].

## 5. The Importance of SPR Biosensors in the Medical Diagnosis 

Many years ago, SPR technology firstly emerged and then many scientists in various fields include chemistry, biology, physics, and medicine have joined to use this promising technology. Recently, SPR biosensors have fascinated impressive attention as medical diagnostic tools due to many reasons. It is easy to prepare them with low cost and without labeling. These sensors provide high specificity and sensitivity, and are capable to detect clinically relevant analyte in real-time. SPR as a simple and direct sensing approach has been used to detect different clinical entities. SPR sensor was used to diagnostic human hepatitis B virus antibodies [27,28]. Additionally, SPR sensor platforms were developed for total prostate-specific antigen detection [29,30]. The extraordinary properties of graphene were exploited to construct SPR sensor to detect folic acid protein [31]. Magnetic nanoparticles with core-shell structure added amplification technique to the SPR sensor to detect α-fetoprotein [32]. The combination of SPR sensor with advantages of molecular imprinting-based synthetic receptors was reported in the detection of cardiac biomarkers used to diagnose acute myocardial infarction such as myoglobin, creatine kinase-myocardial band, and cardiac troponins [33]. SPR chips were modified using lysozyme imprinted polymeric nanoparticles to detect the changes in lysozyme levels, which work as indicators for some diseases including leukemia, meningitis, several kidney problems, and rheumatoid arthritis [34]. It was demonstrated that SPR sensor has the ability to detect pregnancy associated plasma protein A2, which is a metalloproteinase that plays multiple roles in fetal development and postnatal growth [35]. Several studies were reported on using SPR sensor as a new strategy to detect influenza nucleoprotein [36], avian influenza A H7N9 virus [37], maize chlorotic mottle virus [38], herpes simplex virus (HSV) [39], cancer cell line (HeLa cells) with biomarker Rhodamine 6G related to cancer tumors [40], nonstructural protein 1 of Zika virus [41], non-human pathogen feline calicivirus (FCV) [42], and dengue virus (DENV) [43,44,45,46].

SPR technology proved its efficiency again in clinical field as a diagnostic tool of the endocrine diseases. Using the SPR sensor, hormone levels can be directly monitored and measured. By immobilization of the molecularly imprinted nanoparticles onto the SPR chip it was easy to detect iron regulating hormone Hepcidin-25 [47]. Other modified SPR sensors were employed to detect testosterone [48,49], gonadotropic hormones and luteinizing hormone [50], pituitary hormones such as human thyroid-stimulating, growth, follicle-stimulating [51], and insulin [52]. There are other reviews and articles that focus on the importance and the application of SPR biosensors for the diagnosis of medically important entities such as viruses, neurotransmitters, proteins, hormones, nucleic acids, cells, drugs, and disease biomarkers [53,54,55,56,57].

## 6. DA and Its Critical Role in the Human Body

**Figure 5:**
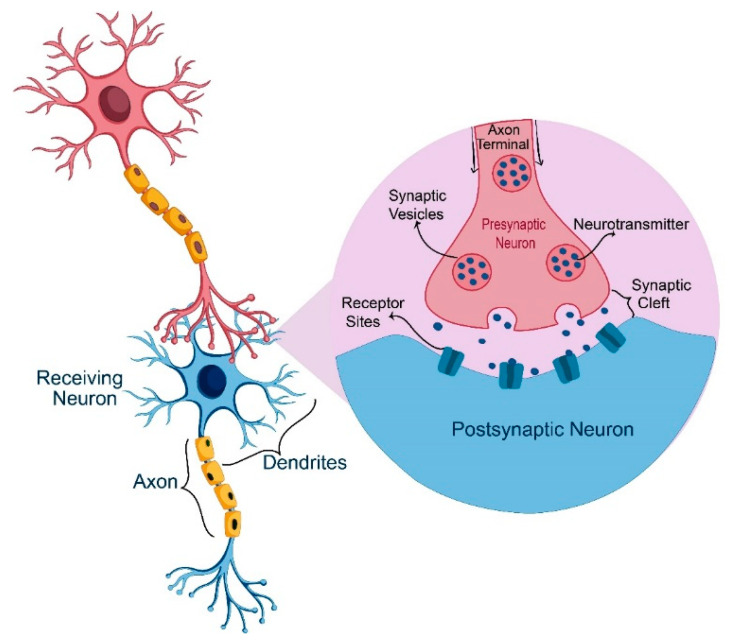
Neuron communication process.

In mammalian central nervous systems, during the synaptic transmission process neurons secrete endogenous chemical messengers that are called neurotransmitters (NTs). NTs transmit signals across synapses from one neuron cell to a target neuron cell as shown in Figure 5 or to muscle cells, gland cells and other non-neuronal body cells. Thus, NTs relay information throughout the brain and the whole-body. DA is one of the most crucially important NTs, it plays a vital role in the neural functions like information flow and attention span, consciousness, learning, motions, emotions and memory formation. In addition to its critical roles related to renal, hormonal and cardiovascular systems [58,59,60,61].

Abnormal concentrations of DA in different biological fluids are associated with various diseases. Cardiotoxicity and subsequent rapid heart rate, hypertension, and heart failure can be an indicator of the high levels of DA [62]. While deficiency or practically complete depletion of DA may result in various neurodegenerative diseases such as Parkinson’s disease (PD) [63,64], Alzheimer’s disease [65,66], schizophrenia [67,68,69,70,71,72,73,74,75], and depression [76]. Therefore, the fabrication and development of highly sensitive and selective sensors for quantitative determination of DA in vivo and in vitro is extremely necessary in the clinical diagnosis, it can make great contribution monitoring the effectiveness of the treatment, prevention of diseases [77]. The monitoring of DA levels can be done in different biological samples including saliva, urine, plasma, serum, platelets, and cerebral spinal fluid [78,79]. DA physiological levels in humans vary in these biofluids. According to the Human Metabolome Database, DA concentration is less than 130 pM in blood, while its levels in human urine and cerebrospinal fluid are approximately 5 nM [80]. To date, the development of analytical methods to measure the concentration of DA directly continues to grow. A variety of these methods demonstrated its capability to detect low levels of DA including high performance liquid chromatography (HPLC) [81,82,83], capillary electrophoresis [84,85,86,87], Fourier transform infrared (FTIR) spectroscopy [88], flow injection [89], enzymatic methods [90], electrochemical (EC) methods [91,92,93], mass spectroscopy [94,95,96], and various types of optical methods such as colorimetry and spectrophotometry [97], fluorescence [98,99,100,101], electrochemiluminescence (ECL) [102], surface-enhanced Raman spectroscopy (SERS) [103,104,105,106,107,108], chemiluminescence (CL) [109], photoelectrochemical (PEC), photoluminescence (PL), solid phase spectrophotometry (SPS), resonance Rayleigh scattering (RRS), and SPR spectroscopy [110,111,112,113,114]. The interested reader in electrochemical and optical methods is referred to the review paper by Kamal Eddin and Fen (2020) [115], and references cited therein.

## 7. DA Detection Using SPR Biosensors

Direct detection using SPR biosensor has the most notable benefit, which is the determination of the kinetics of biomolecular interactions. To study biomolecular interactions using SPR, there is no need to understand the optical phenomena in all its details. It is enough to know that SPR based sensors use an optical method to measure the RI changes near a sensor surface (within 300 nm from the surface). The SPR based sensor does not need complex equipment. During the design of this sensor, the optical unit, the liquid-containing unit and the sensor surface are combined into one system. The main focus in the preceding sections in this review is on SPR physical basics. However, it is necessary also to focus on the sensor chip and its surface chemistry where the biomolecular interactions take place. The few nanometers thickness of the coating and its morphology affects the SPR sensor performance and the quality of the obtained data. The SPR optical sensor had been used to characterize biomolecular interactions qualitatively and quantitatively due to their unparalleled advantages. The unique properties of SPR sensor made it a versatile tool used in various applications such as health care and medical diagnostics [116,117], food control [118,119], environmental pollutant monitoring [120,121,122,123,124,125,126], and others. However, their use to detect NTs is still growing and has attracted the attention of the scientific community.

SPR sensors detect the change in dielectric constants near noble metal film on the sensor chips. Therefore, the concern during the sensor design is on the surface modification and the immobilization of molecular recognition elements such as proteins, DNA, natural, and synthetic polymers to increase the selectivity to the detected analyte. The employment of molecularly imprinted polymer (MIP) with immobilized Au nanoparticles (NPs) (Au-MIP) to SPR sensors as a recognition element during DA sensing was reported by Matsui et al. (2005) [127]. Sensor chip modification was conducted in two steps, firstly the Au substrate was modified with MIP without Au NPs to avoid immobilization of Au NPs in too close vicinity to the Au substrate, which in turn reduces the sensitivity, then it was modified with Au NPs-MIP. The MIP swelling by incorporating water in accordance with analyte binding changes the dielectric constant near the surface of Au substrate significantly. More importantly, the distance between the Au NPs within the polymer gel and Au film on the sensor chip surface would be increased because of this swelling, which enhances the degree of SPR angle shift strongly. The modified sensor chip showed an increasing SPR angle in response to DA concentration in the range (1 nM to 1 mM). Furthermore, the Au NPs effectively enhanced the signal intensity (the change of SPR angle) in comparison with a sensor chip without Au NPs. Temperature-dependent behaviors of the sensor chips was also investigated by measuring SPR angle at different temperature. Au-MIP exhibited swelling in response to the low temperature. The proposed sensor demonstrated its repeatability, this because the analyte binding process and the consequent swelling was reversible.

The sensitivity of the Au-MIP-based SPR sensor can be also emphasized by comparison with the colorimetric sensor using a spectrophotometer [128], in which Au-MIP exhibited significant spectroscopic changes at 5 µM or higher concentrations.

The utilization of natural receptor to develop SPR biosensor was reported by Kumbhat et al. (2007) [129]. They developed an SPR based affinity biosensor using a D_3_-DA receptor (DA-RC) as a recognition molecule for DA detection. During the immobilization of DA with bovine serum albumin (BSA) protein (DA–BSA) conjugate on the sensor chip by physical adsorption, the loss of activity of biomolecules was minimized. The DA–BSA conjugate flowed over the gold surface, the increase in the resonance angle was an indicator on the saturation of the immobilization onto the sensor surface. Different concentrations (5–400 µg/mL) of the conjugate were used in the immobilization. The SPR angle shift reached a plateau around 100 µg/mL of the DA–BSA conjugate. The principle of indirect competitive inhibition to detect DA was employed in this work. DA-RC was allowed to flow over the DA–BSA surface, the resonance angle increased due to the binding of the receptor to the DA haptens present on the sensor surface. After completion of the DA-RC flow, the flow was switched back to phosphate buffered saline (PBS) buffer and the resonance angle shift remained almost stable due to the strong affinity interaction between DA-RC and DA–BSA conjugate. The sensor sensograms were observed for the affinity reaction between immobilized DA–BSA conjugate and DA-RC in the absence of DA, and in the presence of different concentrations of DA. The shift in the resonance angle decreased by increasing in the concentration of free DA in solution, this was because the free DA inhibited the binding interaction of DA-RC with the DA–BSA conjugate. The sensor exhibited a linear detection range from 0.085 to 700 ng/mL with a lower LOD of 0.085 ng/mL. The results showed that there is no significant interference species such as ascorbic acid (AA), uric acid (UA), and other DA analogues viz., DOPAC and 3-(3,4 dihydroxyphenyl)-alanine (DOPA). The stability of the sensor surface was high during repeated regeneration and affinity reaction cycles. According to the simplicity and effectiveness of this biosensor, their study presented an encouraging scope to develop portable detection systems to measure DA in vitro and in vivo in clinical and medical diagnostics.

The adsorption capability of Ibuprofen and DA on pure gold layer and gold functionalized with L-cysteine (L-Cys) and L-glutathione (L-GSH) using SPR spectroscopy was investigated by Sebők et al. (2013) [112]. From the description of DA adsorption by a two-stage isotherm it could be concluded that first layer of DA was irreversibly bound while the second layer was anchored by physical forces, so DA adsorption on the gold surface was only partially reversible. The amino groups available in DA enabled formation of a stronger chemical bond with the cysteine molecule comparing with ibuprofen. The bounded DA on the gold surface functionalized with glutathione showed perpendicular orientation to the gold surface, while glutathione orientation was the same as ibuprofen parallel to the surface. The initial energies of glutathione/ibuprofen interaction were lower comparing with the glutathione/DA system.

Among two-dimensional materials used to design high performance sensor platform, graphene has received profound interest due to its remarkable properties. In the work reported by Kamali et al. (2015), they developed the silver @ graphene oxide (Ag@GO) nanocomposite-based SPR optical sensor to detect DA and other biomolecules such as AA and UA [130]. The nanocomposite showed an SPR band at 402 nm due to the Ag NPs. The SPR intensity-based LOD of DA was 49 nM in the DA concentration linear range (100 nM to 1 µM), and 62 nM in the range (1–2 µM). While the SPR band position-based LOD of DA was 30 nM. The SPR absorbance peak changes toward UA and AA were not comparable with DA, which proved its excellent selectivity. The adsorption and sensing ability of the Ag@GO nanocomposite mainly depended on the nature of the adsorption site and its interaction with the functional groups of the molecules. The results proved that DA had more affinity with Ag@GO than UA and DA.

Next, Rithesh Raj et al. (2016) used green synthesized Ag NPs as sensing material to fabricate SPR based fiber optic sensor for DA measurement [131]. SPR spectra showed decrease in SPR dip intensity. Additionally, a shift in resonance wavelength towards lower wavelength was observed by increasing the concentrations of DA, this probably occurred because of the interaction between DA and Ag clusters which led to formation of Ag NPs. Poly vinyl alcohol (PVA) coated fiber showed negligible shift in wavelength comparing to green Ag NPs coated fiber, which demonstrated the role of the green Ag NPs in the sensing enhancement. The sensor response time was 6 min and the corresponding maximum resonance wavelength shift was 12 nm. The LOD was 2 × 10^−7^ M enhanced the selectivity of the proposed sensor in the presence of other biological species. 

The utilization of conjugated polymer layer consisted of P(NIPAAm-st-MAAmBO) and glycopolymer (poly (2-lactobionamidoethyl methacrylamide)(PLAEMA) to functionalize the SPR sensing platform for DA detection was reported by Jiang et al. (2017) [132]. The SPR sensing mechanism was based on DA induced swelling of the conjugated polymer layer thickness, which increased the local IR and the reflection angle. This reversible swelling allows regeneration of the sensor. The increase in the reflection angle was measured as the sensogram change for quantitative analysis. By changing DA concentration in the range 1–10 nM, the sensogram exhibited a linear relation with DA concentration with a LOD of 1 nM. Increasing DA concentration led to an increase in the rate of adsorption because the multiple binding sites become more available on the P(NIPAAm_149_-st-MAAmBO_19_) chain. This in turn reduces the time of detection. By injection higher concentrations of DA, the binding sites were saturated then the resolution of the conjugated polymer functionalized sensor decreased. The proposed sensor had a broad dynamic range of 10^−9^ to 10^−4^ M.

The selectivity of the sensor was tested by comparing sensogram variation caused by different injections with 5 nM DA, glucose, AA and UA samples using SPR sensors functionalized with conjugated P(NIPAAm_149_-st-MAAmBO_19_) and P(LAEMA_21_); P(NIPAAm_149_-st-MAAmBO_19_) only. The conjugated polymer functionalized sensor was selective to DA only. Additionally, the developed sensor was able to recognizing DA with coexisting saccharides. Polymer functionalized sensor provided much improved stability for at least 2 weeks in comparison with methods using specific DA receptors [133].

In the same year Abd Manaf et al. (2017) presented novel SPR sensor to detect DA down to 50 pM with high sensitivity of 36 nm/nM [134]. They developed a four-layer coating structure including SU8 waveguide, platinum, platinum NPs, and plastic for highly sensitive and selective measurements. Using Pt NPs, the effective surface area efficiently increased and the interaction between coating and waveguide channel increased in comparison with the metal coating without modification. The proposed sensor was designed for operation at high wavelength ranging between 1450 and 1600 nm to get high compatibility with normal optic fibers and achieve low power loss. The partial absorption of the input signal occurred through DA and Pt, and a power loss was noted at the output depending on the refractive indices of Pt and DA. Depending on the properties of the material used, in a certain wavelength, the absorption was at maximum (SPR dip). The RI of the DA solution was measured in order to identify the optimal thickness of the Pt layer using a prism coupler at 1550 nm wavelength. At a thickness below 50 nm of the Pt layer, too low sensitivity was obtained. While no significant improvement was observed when the thickness was more than 50 nm. Therefore, the suitable Pt layer was with a thickness of 50 nm. With changing DA concentration from 1 to 10 nM. When DA concentration decreases, SPR spectra shows shift in the resonance wavelength occurs toward a shorter wavelength. The dip was observed in the range 1500 and 1530 nm of wavelength. The sensor selectivity was high in the presence of glucose, lysine, and AA. The power loss was not significant at long wavelength, which qualifies this sensor to be used in high-precision application.

Recently, Cao and McDermott (2018) reported an ultrasensitive and selective method for DA detection. They incorporate DA DNA aptamer (DAAPT) conjugated AuNP that enhanced the inhibition assay using SPR imaging to detect and quantitate DA down to 200 fM. To the best of our knowledge, this is the lowest LOD achieved for SPR sensing of DA until now [80]. All the spectra have a good shape with the LSPR peaks located around 520 nm. The successful conjugation of DAAPT to AuNP surface was confirmed by observed the red shifts of the LSPR peaks. By mixing the 10 nm DAAPT-AuNP conjugate solution with DA solution at specific concentration, the binding between DA molecules and DAAPT-AuNP conjugates occurred, which in turns decreased the effective concentration of DAAPT-AuNP conjugate that can bind to the complementary DNA (cDNA) probe immobilized on a gold chip. With increasing DA concentrations, all the DAAPTs on the AuNP conjugate surface had the chance to bind to their DA targets so the conjugate probe is in its “OFF” state. In this case, the DNA aptamer was blocked and could not bind to cDNA on the chip surface, thus no signal response was observed. On the contrary, in the absence of DA, the binding between DAAPT on AuNP surface and DA did not happen, then the conjugate probe was in its “ON” state. Additionally, the binding of the conjugate to the cDNA probe was detected strongly. As a result, a big signal response was generated. In this study, the target DA molecule was acting as a switch to turn “OFF” and “ON” the DAAPT-AuNP conjugate during the whole process. The proposed assay showed good sensitivity, reproducibility, and its specificity to DA was high. Using this nanoparticle enhanced SPR aptasensor has the potential to detect small molecules, proteins, and other analytes, as long as the target has a corresponding aptamer.

Additionally, in 2018, Sharma used molecular imprinted graphene nanoplatelets (GNP)/tin oxide (SnO_2_) nanocomposite to fabricate a fiber optic SPR-based DA sensor with high selectivity. The LOD of this sensor was lower than the LOD values of other DA sensors, it was 0.031 µM [135]. The dipping time of sensing element over the silver coated probe greater than 20 min produces sensing layer with a large thickness, which perturbs the intensity of the electric field at the interface and decreases the shift. By increasing DA concentration, the blue shift with a decrease in the depth of the SPR spectra was recorded. The change in the real and imaginary parts of the RI of the sensing layer occurred as a result of the binding of DA molecules with active sites in the sensing layer. The decrease in the real part of the effective RI led to that observed blue shift.

Recently, Sun et al. (2019) proposed an Au film/graphene D-shaped plastic optical fiber (D-POF) functionalized with DA binding aptamer (DBA) to detect DA sensitively and selectively in the presence of three different interferences. They demonstrated that graphene enhanced the sensitivity and played a role in amplification of the SPR signal by introducing DBA. They demonstrated the potential of this sensor to detect DA-induced disorders [136]. Graphene improved the sensor sensitivity and amplified the SPR signal. As RI changed from 1.3330 to 1.3612, the resonant wavelength of the proposed sensor had a red shift of 43.4 nm, which was larger than that of the sensor covered only by Au film 34.9 nm. Adding graphene increased the sensor sensitivity from 1238 to 1539 nm/RIU. The SPR dips decreased as the RI increased due to the higher loss of energy and the increasing in the penetration depth of evanescent field.

In the same year, Yuan et al. used DA to modify a gold sensing surface and produce self-assembled monolayers for secondary surface mediated reactions under different environmental conditions including pH, different buffers, varying DA concentrations in buffer solutions as well as the immobilization time of DA [137]. The favorable environmental conditions for DA immobilization onto gold surface were only alkaline (pH 7.6) and mildly alkaline media (pH 8.6 or 9.5) for 1 h. The immobilization for 2 h appeared to occur only in pH 7.6 or pH 8.6, and the SPR phenomenon did not exist at pH 9.5. This is because the thickness of the immobilized DA was over a critical reflectivity value. Using 2 mg/mL DA in pH 8.6 Tris buffer resulted in the optimum reactive on the gold chip. The critical DA immobilization time for SPR disappearance under the previous favorable conditions was estimated to be 277 min. These results provide valuable information for using DA for surface modification in the future studies. The recent works that used SPR phenomenon in DA measurements are summarized and discussed in Table 1.

## 8. The Advantages of DA Detection Using SPR Sensors

To evaluate the performance of any sensor, it is necessary to focus on some parameters that reveal its validity and efficiency. Among the most important of these parameters are the sensitivity and LOD, which must be sufficient enough for the low concentrations of the target. Additionally, the selectivity is very important to distinguish the target in the real sample where different interfering species exist. Reproducibility and overall reliability are also required. The detection limits of most sensors developed to detect DA were in the micromolar level. However, these sensors are not suitable for clinical diagnostics, which requires a nanomolar level of detection. Over the years, practical and economical SPR sensors have proven their efficiency in several fields as previously mentioned due to their overwhelming advantages. By employing SPR sensors, the reported LODs for DA are typically in the pM range or higher (nM). Recently, the LOD value in the range fM was obtained using this sensor. Table 2 shows a comparison between the lowest detection limit of different DA sensors. Obviously, the lowest LODs were in fM range obtained using EC, SERS, and SPR sensors. Despite the promising LODs obtained using EC and SERS sensors, they still have several disadvantages.

An EC sensor offers real-time detection with simplicity and excellent sensitivity, fast response time, wide linear concentration range, cost effectiveness, and an ability to be miniaturized. However, it suffers from limited selectivity in the presence of other biological analytes, large noise and background signal. In addition to the fouling of the sensor surface and its degradation over time. Although the sensitivity and selectivity of the SERS-based DA sensors are higher in comparison to other detection methods. However, there is an obstacle to the availability of these sensors, they require expensive equipment for analysis. An SPR sensor has several important advantages such as direct label free detection of diverse molecule sets including small molecules, real-time measurements, high reliability, very high sensitivity with low detection limit, long-term stability, cost-effectiveness, easy sample preparation, small consumption of sample and reagent, reproducibility, regeneration of sensor chips, and specificity to the binding event. On the other hand, the disadvantages of this sensor are non-specific binding to surfaces needs to be controlled, which requires a meticulous experimental design, mass transport limitations when the analyte transfer to the ligand is limited, the immobilization effects, and the ligand can change its orientation after immobilization on the sensor chip and prevents binding with the analyte [151]. The combination of SERS with SPR has the potential to massively increase the local electromagnetic field intensity of nanoparticles to a level far exceeding the single-molecule SERS detection limit but this still lacks versatility [152]. However, in the context of a point-of-care testing (POCT) configuration, the bulky nature of the SPR apparatus hinders its field applications. So, the efforts are mainly made on the miniaturization of SPR devices.

## 9. Conclusions

In recent decades, the development of biosensors to identify and measure low concentrations of different NTs with high selectivity, sensitivity, low-cost, and rapid response attracted considerable attention due to the crucial role that neurotransmitters play in clinical diagnostics and curing mental disorders such as schizophrenia, Parkinson’s disease, and Alzheimer’s disease. SPR has emerged as a very suitable technology for clinical analytes detection. It is based on IR variations due to the mass change at the sensor chip. The SPR sensing platform has many features. It is easy to prepare it, the basic optics that it needs can be miniaturized to a suitable size in diagnosis, the direct quantification of the specific bindings occurs with high specificity and sensitivity. This low-cost technology is not based on labeling of the target molecules and does not alter its binding affinity and kinetics properties. Rapid and reliable detection of various medically important entities using SPR spectroscopy has been done over the last years. The reported works on DA sensing using this promising method is still limited despite the impressive results obtained. This is a strong motivation for researchers to develop these sensors by modification SPR chips using nanomaterials. Ongoing research employing the unique capabilities of carbon-based nanomaterials as well as the advantages of polymers and incorporating them with SPR technology for ultra-sensitive and selective detection of DA may introduce exciting progress in neuroscience. The high potential of SPR sensors and the continuous efforts to develop them and overcome their limitations qualify them to have a prominent presence in future developments in lab-on-a-chip technologies including point-of-care devices.

## Figures and Tables

**Table 1:** SPR sensors for dopamine (DA) detection.

Material	LOD	Detection Range	References
MIP-Au electrode	1 pM	-	[110]
DA-RC	0.085 ng/mL	0.085 ng/mL–700 ng/mL	[129]
DA antibodies/Au NPs/ITO	1 nM	0.001–100 µM	[113]
Ag@GO	30 nM	100 nM–2 µM	[130]
Ag NPs	0.2 µM	0.2–30 µM	[131]
Conjugated polymer P(NIPAAm149-st-MAAmBO19) and P(LAEMA21)	1 nM	1 nM–0.1 mM	[132]
Pt	50 pM	0.1 nM–32 µM	[134]
DAAPT-AuNPs	200 fM	100 µM–2 mM 200 fM–20 nM	[80]
Molecular Imprinted GNP/SnO_2_ Nanocomposite	31 nM	0–100 µM	[135]
Au/graphene/DBA D-POF	-	0.1 nM–1 µM	[136]

**Table 2:** Comparison of the detection limits of various DA sensors.

Method	Lowest Detection Limit	References
EC	78 fM	[138]
CL	0.19 nM	[139]
ECL	0.31 pM	[140]
Fluorescence	0.1 pM	[141]
Spectrophotometry	0.4 nM	[142]
Colorimetry	0.16 nM	[143]
SERS	0.006 pM	[144]
RRS	0.392 ng/mL	[145]
PRRS	0.1 pM	[146]
SPS	1.7 µM	[147]
PL	10 nM	[148]
Absorption	1.2 nM	[149]
PEC	2.3 pM	[150]
SPR	200 fM	[80]

E—Electrochemical; CL—Chemiluminescence; ECL—Electrochemiluminescence; SERS—Surface-enhanced Raman spectroscopy; RRS—Resonance Rayleigh scattering; PRRS—Plasmonic resonance Rayleigh scattering; SPS—Solid phase spectrophotometry; PL—Photoluminescence; PEC—Photoelectrochemical; SPR—Surface plasmon resonance.

## References

[1] Kan X., Li S.F.Y. (2016). Rapid Detection of Bacteria in Food by Surface Plasmon Resonance Sensors. Int. J. Adv. Sci. Eng. Technol..

[2] Yanase Y., Hiragun T., Ishii K., Kawaguchi T., Yanase T., Kawai M., Sakamoto K., Hide M. (2014). Surface Plasmon Resonance for Cell-Based Clinical Diagnosis. Sensors.

[3] Yanase Y., Sakamoto K., Kobayashi K., Hide M. (2016). Diagnosis of Immediate-Type Allergy Using Surface Plasmon Resonance. Opt. Mater. Express.

[4] Situ C., Mooney M.H., Elliott C.T., Buijs J. (2010). Advances in Surface Plasmon Resonance Biosensor Technology towards High-Throughput, Food-Safety Analysis. TrAC Trends Anal. Chem..

[5] Daniyal W.M.E.M.M., Fen Y.W., Abdullah J., Omar N.A.S., Anas N.A.A., Ramdzan M., Syahira N. (2019). Highly Sensitive Surface Plasmon Resonance Optical Sensor for Detection of Copper, Zinc, and Nickel Ions. Sens. Lett..

[6] Holzinger M., Goff A.L., Cosnier S. (2014). Nanomaterials for Biosensing Applications: A Review. Front. Chem..

[7] Ishimaru A., Jaruwatanadilok S., Kuga Y. (2006). Generalized Surface Plasmon Resonance Sensors Using Metamaterials and Negative Index Materials. Prog. Electromagn. Res..

[8] Pitarke J.M., Silkin V.M., Chulkov E.V., Echenique P.M. (2007). Theory of Surface Plasmons and Surface-Plasmon Polaritons. Rep. Prog. Phys..

[9] Homola J., Yee S.S., Gauglitz G. (1999). Surface Plasmon Resonance Sensors. Sens. Actuators B Chem..

[10] Homola J. (2008). Surface Plasmon Resonance Sensors for Detection of Chemical and Biological Species. Chem. Rev..

[11] Wood R.W. (1902). On a Remarkable Case of Uneven Distribution of Light in A Diffraction Grating Spectrum. Proc. Phys. Soc..

[12] Fano U. (1941). The Theory of Anomalous Diffraction Gratings and of Quasi-Stationary Waves on Metallic Surfaces (Sommerfeld’s Waves). JOSA.

[13] OTTO A. (1968). Excitation of Nonradiative Surface Plasma Waves in Silver by the Method of Frustrated Total Reflection. Z. Phys. A Hadron. Nucl..

[14] Kretschmann E., Raether H. (1968). Radiative Decay of Non Radiative Surface Plasmons Excited by Light. Z. Naturforsch. A Phys. Sci..

[15] Rizal C. (2016). Bio-Magnetoplasmonics, Emerging Biomedical Technologies and Beyond. J. Nanomed. Res..

[16] Fen Y.W., Yunus W.M.M. (2013). Surface Plasmon Resonance Spectroscopy as An Alternative for Sensing Heavy Metal Ions: A Review. Sens. Rev..

[17] Homola J., Koudela I., Yee S.S. (1999). Surface Plasmon Resonance Sensors Based on Diffraction Gratings and Prism Couplers: Sensitivity Comparison. Sens. Actuators B Chem..

[18] Karlssonz R., Fält A. (1997). Experimental Design for Kinetic Analysis of Protein-Protein Interactions with Surface Plasmon Resonance Biosensors. J. Immunol. Methods.

[19] Homola J. (2006). Electromagnetic Theory of Surface Plasmons.

[20] Mukhtar W.M., Halim R.M., Hassan H. (2017). Optimization of SPR Signals: Monitoring the Physical Structures and Refractive Indices of Prisms. EPJ Web Conf..

[21] Al-qazwini Y., Noor A.S.M., Arasu P.T., Sadrolhosseini A.R. (2013). Investigation of the Performance of an SPR-Based Optical Fiber Sensor Using Finite-Difference Time Domain. Curr. Appl. Phys..

[22] Murat N.F., Mukhtar W.M., Rashid A.R.A., Dasuki K.A., Yussuf A.A.R.A. Optimization of Gold Thin Films Thicknesses in Enhancing SPR Response. Proceedings of the 2016 IEEE International Conference on Semiconductor Electronics (ICSE).

[23] Michel D., Xiao F., Alameh K. (2017). A Compact, Flexible Fiber-Optic Surface Plasmon Resonance Sensor with Changeable Sensor Chips. Sens. Actuators B Chem..

[24] Nelson B.P., Frutos A.G., Brockman J.M., Corn R.M. (1999). Near-Infrared Surface Plasmon Resonance Measurements of Ultrathin Films. 1. Angle Shift and SPR Imaging Experiments. Anal. Chem..

[25] Patskovsky S., Kabashin A.V., Meunier M., Luong J.H.T. (2003). Properties and Sensing Characteristics of Surface-Plasmon Resonance in Infrared Light. J. Opt. Soc. Am. A.

[26] Mukhtar W.M., Murat N.F., Samsuri N.D., Dasuki K.A. (2018). Maximizing the Response of SPR Signal: A Vital Role of Light Excitation Wavelength. AIP Conf. Proc..

[27] Chung J.W., Kim S.D., Bernhardt R., Pyun J.C. (2005). Application of SPR Biosensor for Medical Diagnostics of Human Hepatitis B Virus (HHBV). Sens. Actuators B Chem..

[28] Uzun L., Say R., Ünal S., Denizli A. (2009). Production of Surface Plasmon Resonance Based Assay Kit for Hepatitis Diagnosis. Biosens. Bioelectron..

[29] Uludag Y., Tothill I.E. (2012). Cancer Biomarker Detection in Serum Samples Using Surface Plasmon Resonance and Quartz Crystal Microbalance Sensors with Nanoparticle Signal Amplification. Anal. Chem..

[30] Ertürk G., Özen H., Tümer M.A., Mattiasson B., Denizli A. (2016). Microcontact Imprinting Based Surface Plasmon Resonance (SPR) Biosensor for Real-Time and Ultrasensitive Detection of Prostate Specific Antigen (PSA) from Clinical Samples. Sens. Actuators B Chem..

[31] He L., Pagneux Q., Larroulet I., Serrano A.Y., Pesquera A., Zurutuza A., Mandler D., Boukherroub R., Szunerits S. (2017). Label-Free Femtomolar Cancer Biomarker Detection in Human Serum Using Graphene-Coated Surface Plasmon Resonance Chips. Biosens. Bioelectron..

[32] Liang R.P., Yao G.H., Fan L.X., Qiu J.D. (2012). Magnetic Fe 3O 4@Au Composite-Enhanced Surface Plasmon Resonance for Ultrasensitive Detection of Magnetic Nanoparticle-Enriched α-Fetoprotein. Anal. Chim. Acta.

[33] Osman B., Uzun L., Beşirli N., Denizli A. (2013). Microcontact Imprinted Surface Plasmon Resonance Sensor for Myoglobin Detection. Mater. Sci. Eng. C.

[34] Sener G., Uzun L., Say R., Denizli A. (2011). Use of Molecular Imprinted Nanoparticles as Biorecognition Element on Surface Plasmon Resonance Sensor. Sens. Actuators B Chem..

[35] Bocková M., Chadtová Song X., Gedeonová E., Levová K., Kalousová M., Zima T., Homola J. (2016). Surface Plasmon Resonance Biosensor for Detection of Pregnancy Associated Plasma Protein A2 in Clinical Samples. Anal. Bioanal. Chem..

[36] Brun A.P.L., Soliakov A., Shah D.S.H., Holt S.A., Mcgill A., Lakey J.H. (2015). Engineered Self-Assembling Monolayers for Label Free Detection of Influenza Nucleoprotein. Biomed. Microdevices.

[37] Chang Y.F., Wang W.H., Hong Y.W., Yuan R.Y., Chen K.H., Huang Y.W., Lu P.L., Chen Y.H., Chen Y.M.A., Su L.C. (2018). Simple Strategy for Rapid and Sensitive Detection of Avian Influenza A H7N9 Virus Based on Intensity-Modulated SPR Biosensor and New Generated Antibody. Anal. Chem..

[38] Zeng C., Huang X., Xu J., Li G., Ma J., Ji H.F., Zhu S., Chen H. (2013). Rapid and Sensitive Detection of Maize Chlorotic Mottle Virus Using Surface Plasmon Resonance-Based Biosensor. Anal. Biochem..

[39] Cairns T.M., Ditto N.T., Atanasiu D., Lou H., Brooks B.D., Saw W.T., Eisenberg R.J., Cohen G.H. (2019). Surface Plasmon Resonance Reveals Direct Binding of Herpes Simplex Virus Glycoproteins GH/GL to GD and Locates a GH/GL Binding Site on GD. J. Virol..

[40] Firdous S., Anwar S., Rafya R. (2018). Development of Surface Plasmon Resonance (SPR) Biosensors for Use in the Diagnostics of Malignant and Infectious Diseases. Laser Phys. Lett..

[41] Takemura K., Adegoke O., Suzuki T., Park E.Y. (2019). A Localized Surface Plasmon Resonance-Amplified Immunofluorescence Biosensor for Ultrasensitive and Rapid Detection of Nonstructural Protein 1 of Zika Virus. PLoS ONE.

[42] Yakes B.J., Papafragkou E., Conrad S.M., Neill J.D., Ridpath J.F., Burkhardt W., Kulka M., DeGrasse S.L. (2013). Surface Plasmon Resonance Biosensor for Detection of Feline Calicivirus, a Surrogate for Norovirus. Int. J. Food Microbiol..

[43] Omar N.A.S., Fen Y.W., Abdullah J., Chik C.E.N.C.E., Mahdi M.A. (2018). Development of an Optical Sensor Based on Surface Plasmon Resonance Phenomenon for Diagnosis of Dengue Virus E-Protein. Sens. Bio-Sens. Res..

[44] Omar N.A.S., Fen Y.W., Abdullah J., Zaid M.H.M., Mahdi M.A. (2018). Structural, Optical and Sensing Properties of CdS-NH2GO Thin Film as a Dengue Virus E-Protein Sensing Material. Optik.

[45] Omar N.A.S., Fen Y.W., Abdullah J., Mustapha Kamil Y., Daniyal W.M.E.M.M., Sadrolhosseini A.R., Mahdi M.A. (2020). Sensitive Detection of Dengue Virus Type 2 E-Proteins Signals Using Self-Assembled Monolayers/Reduced Graphene Oxide-PAMAM Dendrimer Thin Film-SPR Optical Sensor. Sci. Rep..

[46] Omar N.A.S., Fen Y.W., Abdullah J., Sadrolhosseini A.R., Mustapha Kamil Y., Fauzi N.I.M., Hashim H.S., Mahdi M.A. (2020). Quantitative and Selective Surface Plasmon Resonance Response Based on a Reduced Graphene Oxide–Polyamidoamine Nanocomposite for Detection of Dengue Virus E-Proteins. Nanomaterials.

[47] Cenci L., Andreetto E., Vestri A., Bovi M., Barozzi M., Iacob E., Busato M., Castagna A., Girelli D., Bossi A.M. (2015). Surface Plasmon Resonance Based on Molecularly Imprinted Nanoparticles for the Picomolar Detection of the Iron Regulating Hormone Hepcidin-25. J. Nanobiotechnol..

[48] Zhang Q., Jing L., Wang Y., Zhang J., Ren Y., Wang Y., Wei T., Liedberg B. (2014). Surface Plasmon Resonance Sensor for Femtomolar Detection of Testosterone with Water-Compatible Macroporous Molecularly Imprinted Film. Anal. Biochem..

[49] Yockell-Lelièvre H., Bukar N., McKeating K.S., Arnaud M., Cosin P., Guo Y., Dupret-Carruel J., Mougin B., Masson J.F. (2015). Plasmonic Sensors for the Competitive Detection of Testosterone. Analyst.

[50] Treviño J., Calle A., Rodríguez-Frade J.M., Mellado M., Lechuga L.M. (2009). Single- and Multi-Analyte Determination of Gonadotropic Hormones in Urine by Surface Plasmon Resonance Immunoassay. Anal. Chim. Acta.

[51] Treviño J., Calle A., Rodríguez-Frade J.M., Mellado M., Lechuga L.M. (2009). Surface Plasmon Resonance Immunoassay Analysis of Pituitary Hormones in Urine and Serum Samples. Clin. Chim. Acta.

[52] Sanghera N., Anderson A., Nuar N., Xie C., Mitchell D., Klein-Seetharaman J. (2017). Insulin Biosensor Development: A Case Study. Int. J. Parallel Emerg. Distrib. Syst..

[53] Wang S., Shan X., Patel U., Huang X., Lu J., Li J., Tao N. (2010). Label-Free Imaging, Detection, and Mass Measurement of Single Viruses by Surface Plasmon Resonance. Proc. Natl. Acad. Sci. USA.

[54] Masson J.F. (2017). Surface Plasmon Resonance Clinical Biosensors for Medical Diagnostics. ACS Sens..

[55] Siedhoff D., Strauch M., Shpacovitch V., Merhof D. Unsupervised Data Analysis for Virus Detection with a Surface Plasmon Resonance Sensor. Proceedings of the 2017 Seventh International Conference on Image Processing Theory, Tools and Applications (IPTA).

[56] Victoria S. (2012). Application of Surface Plasmon Resonance (SPR) for the Detection of Single Viruses and Single Biological Nano-Objects. J. Bacteriol. Parasitol..

[57] Saylan Y., Yilmaz F., Özgür E., Derazshamshir A., Bereli N., Yavuz H., Denizli A., Kumar C.S.S.R. (2018). Nanotechnology Characterization Tools for Biosensing and Medical Diagnosis. Surface Plasmon Resonance Sensors for Medical Diagnosis.

[58] Moon J.M., Thapliyal N., Hussain K.K., Goyal R.N., Shim Y.B. (2018). Conducting Polymer-Based Electrochemical Biosensors for Neurotransmitters: A Review. Biosens. Bioelectron..

[59] Soleymani J. (2015). Advanced Materials for Optical Sensing and Biosensing of Neurotransmitters. TrAC Trends Anal. Chem..

[60] Krishna V.M., Somanathan T., Manikandan E., Tadi K.K., Uvarajan S. (2018). Neurotransmitter Dopamine Enhanced Sensing Detection Using Fibre-Like Carbon Nanotubes by Chemical Vapor Deposition Technique. J. Nanosci. Nanotechnol..

[61] Lin X., Zhang Y., Chen W., Wu P. (2007). Electrocatalytic Oxidation and Determination of Dopamine in the Presence of Ascorbic Acid and Uric Acid at a Poly (p -Nitrobenzenazo Resorcinol) Modified Glassy Carbon Electrode. Sens. Actuators B Chem..

[62] Liu J., Wang X., Cui M., Lin L., Jiang S., Jiao L., Zhang L. (2013). A Promising Non-Aggregation Colorimetric Sensor of AuNRs–Ag + for Determination of Dopamine. Sens. Actuators B Chem..

[63] Haven N. (1997). Dopamine Synthesis, Uptake, Metabolism, and Receptors: Relevance to Gene Therapy of Parkinson’s Disease. Exp. Neurol..

[64] Kim J.-H., Auerbach J.M., Rodríguez-Gómez J.A., Velasco I., Gavin D., Lumelsky N., McKay R. (2002). Dopamine neurons derived from embryonic stem cells function in an animal model of Parkinson’s disease. Nature.

[65] Pezzella A., Ischia M., Napolitano A., Misuraca G., Prota G. (1997). Iron-Mediated Generation of the Neurotoxin 6-Hydroxydopamine Quinone by Reaction of Fatty Acid Hydroperoxides with Dopamine: A Possible Contributory Mechanism for Neuronal Degeneration in Parkinson’s Disease. J. Med. Chem..

[66] Hyman B., Van Hoesen G., Damasio A., Barnes C. (1984). Alzheimer’s disease: Cell-specific pathology isolates the hippocampal formation. Science.

[67] Wightman M., May L.J., Michael A.C. (1988). Detection of Dopamine Dynamics in the Brain. Anal. Chem..

[68] Kesby J.P. (2018). Dopamine, Psychosis and Schizophrenia: The Widening Gap between Basic and Clinical Neuroscience. Transl. Psychiatry.

[69] Pandey P.C., Chauhan D.S., Singh V. (2012). Effect of Processable Polyindole and Nanostructured Domain on the Selective Sensing of Dopamine. Mater. Sci. Eng. C.

[70] Yu C., Yan J., Tu Y. (2011). Electrochemiluminescent Sensing of Dopamine Using CdTe Quantum Dots Capped with Thioglycolic Acid and Supported with Carbon Nanotubes. Microchim. Acta.

[71] Shankaran D.R., Iimura K., Kato T. (2003). Simultaneous Determination of Ascorbic Acid and Dopamine at Sol–Gel Composite Electrode. Sens. Actuators B Chem..

[72] Kurzatkowska K., Dolusic E., Dehaen W., Sieron K., Radecka H. (2009). Gold Electrode Incorporating Corrole as an Ion-Channel Mimetic Sensor for Determination of Dopamine. Anal. Chem..

[73] Lin L., Qiu P., Yang L. (2006). Determination of Dopamine in Rat Striatum by Microdialysis and High-Performance Liquid Chromatography with Electrochemical Detection on a Functionalized Multi-Wall Carbon Nanotube Electrode. Anal. Bioanal. Chem..

[74] Zhang L., Lin X. (2005). Electrochemical Behavior of a Covalently Modified Glassy Carbon Electrode with Aspartic Acid and Its Use for Voltammetric Differentiation of Dopamine and Ascorbic Acid. Anal. Bioanal. Chem..

[75] Jagadeesh J.S., Natarajan S. (2013). Schizophrenia: Interaction between Dopamine, Serotonin, Glutamate, GABA. RJPBCS.

[76] Davis K.L., Kahn R.S., Ko G., Davidson M. (1991). Dopamine in schizophrenia: A review and reconceptualization. Am. J. Psychiatry.

[77] Rui Z., Huang W., Chen Y., Zhang K., Cao Y., Tu J. (2017). Facile Synthesis of Graphene / Polypyrrole 3D Composite for a High-Sensitivity Non-Enzymatic Dopamine Detection. J. Appl. Polym. Sci..

[78] Roy A., Pickar D., De Jong J., Karoum F., Linnoila M. (1988). Norepinephrine and its metabolites in cerebrospinal fluid, plasma, and urine: Relationship to hypothalamic-pituitary-adrenal axis function in depression. Arch. Gen. Psychiatry.

[79] Okumura T., Nakajima Y., Matsuoka M., Takamatsu T. (1997). Study of Salivary Catecholamines Using Fully Automated Column-Switching High-Performance Liquid Chromatography. J. Chromatogr. B Biomed. Appl..

[80] Cao Y., Mcdermott M.T. (2018). Femtomolar and Selective Dopamine Detection by a Gold Nanoparticle Enhanced Surface Plasmon Resonance Aptasensor. bioRxiv.

[81] Yoshitake T., Yoshitake S., Fujino K., Nohta H., Yamaguchi M., Kehr J. (2004). High-Sensitive Liquid Chromatographic Method for Determination of Neuronal Release of Serotonin, Noradrenaline and Dopamine Monitored by Microdialysis in the Rat Prefrontal Cortex. J. Neurosci. Methods.

[82] Carrera V., Sabater E., Vilanova E., Sogorb M.A. (2007). A Simple and Rapid HPLC-MS Method for the Simultaneous Determination of Epinephrine, Norepinephrine, Dopamine and 5-Hydroxytryptamine: Application to the Secretion of Bovine Chromaffin Cell Cultures. J. Chromatogr. B Anal. Technol. Biomed. Life Sci..

[83] Muzzi C., Bertocci E., Terzuoli L., Porcelli B., Ciari I., Pagani R., Guerranti R. (2008). Simultaneous Determination of Serum Concentrations of Levodopa, Dopamine, 3-O-Methyldopa and α-Methyldopa by HPLC. Biomed. Pharmacother..

[84] Woolley A.T., Lao K., Glazer A.N., Mathies R.A. (1998). Capillary Electrophoresis Chips with Integrated Electrochemical Detection. Anal. Chem..

[85] Wang L., Liu Y., Xie H., Fu Z. (2012). Trivalent Copper Chelate-Luminol Chemiluminescence System for Highly Sensitive CE Detection of Dopamine in Biological Sample after Clean-up Using SPE. Electrophoresis.

[86] Zhao Y., Zhao S., Huang J., Ye F. (2011). Quantum Dot-Enhanced Chemiluminescence Detection for Simultaneous Determination of Dopamine and Epinephrine by Capillary Electrophoresis. Talanta.

[87] Thabano J.R.E., Breadmore M.C., Hutchinson J.P., Johns C., Haddad P.R. (2009). Silica Nanoparticle-Templated Methacrylic Acid Monoliths for in-Line Solid-Phase Extraction-Capillary Electrophoresis of Basic Analytes. J. Chromatogr. A.

[88] Wang X., Jin B., Lin X. (2002). In-Situ FTIR Spectroelectrochemical Study of Dopamine at a Glassy Carbon Electrode in a Neutral Solution. Anal. Sci..

[89] Abaidur S.M., Alothman Z.A., Alam S.M., Lee S.H. (2012). Flow Injection-Chemiluminescence Determination of Dopamine Using Potassium Permanganate and Formaldehyde System. Spectrochim. Acta Part A Mol. Biomol. Spectrosc..

[90] Fritzen-Garcia M.B., Monteiro F.F., Cristofolini T., Acuña J.J.S., Zanetti-Ramos B.G., Oliveira I.R.W.Z., Soldi V., Pasa A.A., Creczynski-Pasa T.B. (2013). Characterization of Horseradish Peroxidase Immobilized on PEGylated Polyurethane Nanoparticles and Its Application for Dopamine Detection. Sens. Actuators B Chem..

[91] Liu S., Sun W., Hu F. (2012). Graphene Nano Sheet-Fabricated Electrochemical Sensor for the Determination of Dopamine in the Presence of Ascorbic Acid Using Cetyltrimethylammonium Bromide as the Discriminating Agent. Sens. Actuators B Chem..

[92] Sajid M., Nazal M.K., Mansha M., Alsharaa A., Jillani S.M.S., Basheer C. (2016). Chemically Modified Electrodes for Electrochemical Detection of Dopamine in the Presence of Uric Acid and Ascorbic Acid: A Review. TrAC Trends Anal. Chem..

[93] Shin J.-W., Kim K.-J., Yoon J., Jo J., El-Said W.A., Choi J.-W. (2017). Silver Nanoparticle Modified Electrode Covered by Graphene Oxide for the Enhanced Electrochemical Detection of Dopamine. Sensors.

[94] Hows M.E.P., Lacroix L., Heidbreder C., Organ A.J., Shah A.J. (2004). High-Performance Liquid Chromatography/Tandem Mass Spectrometric Assay for the Simultaneous Measurement of Dopamine, Norepinephrine, 5-Hydroxytryptamine and Cocaine in Biological Samples. J. Neurosci. Methods.

[95] Moini M., Schultz C.L., Mahmood H. (2003). CE/Electrospray Ionization-MS Analysis of Underivatized D/L-Amino Acids and Several Small Neurotransmitters at Attomole Levels through the Use of 18-Crown-6-Tetracarboxylic Acid as a Complexation Reagent/Background Electrolyte. Anal. Chem..

[96] Syslová K., Rambousek L., Kuzma M., Najmanová V., Bubeníková-Valešová V., Šlamberová R., Kačer P. (2011). Monitoring of Dopamine and Its Metabolites in Brain Microdialysates: Method Combining Freeze-Drying with Liquid Chromatography-Tandem Mass Spectrometry. J. Chromatogr. A.

[97] Reza Hormozi Nezhad M., Tashkhourian J., Khodaveisi J., Reza Khoshi M. (2010). Simultaneous Colorimetric Determination of Dopamine and Ascorbic Acid Based on the Surface Plasmon Resonance Band of Colloidal Silver Nanoparticles Using Artificial Neural Networks. Anal. Methods.

[98] Wang H.Y., Hui Q.S., Xu L.X., Jiang J.G., Sun Y. (2003). Fluorimetric Determination of Dopamine in Pharmaceutical Products and Urine Using Ethylene Diamine as the Fluorigenic Reagent. Anal. Chim. Acta.

[99] Kruss S., Landry M.P., Vander Ende E., Lima B.M.A., Reuel N.F., Zhang J., Nelson J., Mu B., Hilmer A., Strano M. (2014). Neurotransmitter Detection Using Corona Phase Molecular Recognition on Fluorescent Single-Walled Carbon Nanotube Sensors. J. Am. Chem. Soc..

[100] Zhao F., Kim J. (2015). Fabrication of a Dopamine Sensor Based on Carboxyl Quantum Dots. J. Nanosci. Nanotechnol..

[101] Kruss S., Salem D.P., Vuković L., Lima B., Vander Ende E., Boyden E.S., Strano M.S. (2017). High-Resolution Imaging of Cellular Dopamine Efflux Using a Fluorescent Nanosensor Array. Proc. Natl. Acad. Sci. USA.

[102] Qi H., Peng Y., Gao Q., Zhang C. (2009). Applications of Nanomaterials in Electrogenerated Chemiluminescence Biosensors. Sensors.

[103] Bu Y., Lee S. (2012). Influence of Dopamine Concentration and Surface Coverage of Au Shell on the Optical Properties of Au, Ag, and Ag CoreAu Shell Nanoparticles. ACS Appl. Mater. Interfaces.

[104] Bu Y., Lee S.-W. (2013). Optical Properties of Dopamine Molecules with Silver Nanoparticles as Surface-Enhanced Raman Scattering (SERS) Substrates at Different PH Conditions. J. Nanosci. Nanotechnol..

[105] Ranc V., Markova Z., Hajduch M., Prucek R., Kvitek L., Kaslik J., Safarova K., Zboril R. (2014). Magnetically Assisted Surface-Enhanced Raman Scattering Selective Determination of Dopamine in an Artificial Cerebrospinal Fluid and a Mouse Striatum Using Fe_3_O_4_/Ag Nanocomposite. Anal. Chem..

[106] An J.H., Choi D.K., Lee K.J., Choi J.W. (2015). Surface-Enhanced Raman Spectroscopy Detection of Dopamine by DNA Targeting Amplification Assay in Parkisons’s Model. Biosens. Bioelectron..

[107] Wang P., Xia M., Liang O., Sun K., Cipriano A.F., Schroeder T., Liu H., Xie Y.H. (2015). Label-Free SERS Selective Detection of Dopamine and Serotonin Using Graphene-Au Nanopyramid Heterostructure. Anal. Chem..

[108] Lu J., Xu C., Nan H., Zhu Q., Qin F., Manohari A.G., Wei M., Zhu Z., Shi Z., Ni Z. (2016). SERS-Active ZnO/Ag Hybrid WGM Microcavity for Ultrasensitive Dopamine Detection. Appl. Phys. Lett..

[109] Deftereos N.T., Calokerinos A.C., Efstathiou C.E. (1993). Flow Injection Chemiluminometric Determination of Epinephrine, Norepinephrine, Dopamine and L-DOPA. Analyst.

[110] Dutta P., Pernites R.B., Danda C., Advincula R.C. (2011). SPR Detection of Dopamine Using Cathodically Electropolymerized, Molecularly Imprinted Poly-p-Aminostyrene Thin Films. Macromol. Chem. Phys..

[111] Jia K., Khaywah M.Y., Li Y., Bijeon J.L., Adam P.M., Déturche R., Guelorget B., François M., Louarn G., Ionescu R.E. (2014). Strong Improvements of Localized Surface Plasmon Resonance Sensitivity by Using Au/Ag Bimetallic Nanostructures Modified with Polydopamine Films. ACS Appl. Mater. Interfaces.

[112] Sebők D., Csapó E., Preočanin T., Bohus G., Kallay N., Dékány I. (2013). Adsorption of Ibuprofen and Dopamine on Functionalized Gold Using Surface Plasmon Resonance Spectroscopy at Solid-Liquid Interface. Croat. Chem. Acta.

[113] Choi J.-H., Lee J.-H., Oh B.-K., Choi J.-W. (2014). Localized Surface Plasmon Resonance-Based Label-Free Biosensor for Highly Sensitive Detection of Dopamine. J. Nanosci. Nanotechnol..

[114] Su R., Pei Z., Huang R., Qi W., Wang M., Wang L., He Z. (2015). Polydopamine-Assisted Fabrication of FiberOptic Localized Surface Plasmon Resonance Sensor Based on Gold Nanoparticles. Trans. Tianjin Univ..

[115] Kamal Eddin F.B., Fen Y.W. (2020). Recent Advances in Electrochemical and Optical Sensing of Dopamine. Sensors.

[116] Omar N.A.S., Fen Y.W. (2018). Recent Development of SPR Spectroscopy as Potential Method for Diagnosis of Dengue Virus E-Protein. Sens. Rev..

[117] Omar N.A.S., Fen Y.W., Saleviter S., Daniyal W.M.E.M.M., Anas N.A.A., Ramdzan N.S.M., Roshidi M.D.A. (2019). Development of a Graphene-Based Surface Plasmon Resonance Optical Sensor Chip for Potential Biomedical Application. Materials.

[118] Zainuddin N.H., Fen Y.W., Alwahib A.A., Yaacob M.H., Bidin N., Omar N.A.S., Mahdi M.A. (2018). Detection of Adulterated Honey by Surface Plasmon Resonance Optical Sensor. Optik.

[119] Sadrolhosseini A.R., Rashid S.A., Jamaludin N., Noor A.S.M. (2019). Surface Plasmon Resonance Sensor Using Polypyrrole-Chitosan/Graphene Quantum Dots Layer for Detection of Sugar. Mater. Res. Express.

[120] Roshidi M.D.A., Fen Y.W., Daniyal W.M.E.M.M., Omar N.A.S., Zulholinda M. (2019). Structural and Optical Properties of Chitosan–Poly(Amidoamine) Dendrimer Composite Thin Film for Potential Sensing Pb^2+^ Using an Optical Spectroscopy. Optik.

[121] Daniyal W.M.E.M.M., Fen Y.W., Abdullah J., Sadrolhosseini A.R., Saleviter S., Omar N.A.S. (2019). Label-Free Optical Spectroscopy for Characterizing Binding Properties of Highly Sensitive Nanocrystalline Cellulose-Graphene Oxide Based Nanocomposite towards Nickel Ion. Spectrochim. Acta A.

[122] Roshidi M.D.A., Fen Y.W., Omar N.A.S., Saleviter S., Daniyal W.M.E.M.M. (2019). Optical Studies of Graphene Oxide/Poly(Amidoamine) Dendrimer Composite Thin Film and Its Potential for Sensing Hg^2+^ Using Surface Plasmon Resonance Spectroscopy. Sens. Mater..

[123] Zainudin A.A., Fen Y.W., Yusof N.A., Al-Rekabi S.H., Mahdi M.A., Omar N.A.S. (2018). Incorporation of Surface Plasmon Resonance with Novel Valinomycin Doped Chitosan-Graphene Oxide Thin Film for Sensing Potassium Ion. Spectrochim. Acta A.

[124] Sadrolhosseini A.R., Naseri M., Rashid S.A. (2017). Polypyrrole-Chitosan/Nickel-Ferrite Nanoparticle Composite Layer for Detecting Heavy Metal Ions Using Surface Plasmon Resonance Technique. Opt. Laser Technol..

[125] Alwahib A.A., Sadrolhosseini A.R., An’Amt M.N., Lim H.N., Yaacob M.H., Abu Bakar M.H., Ming H.N., Mahdi M.A. (2016). Reduced Graphene Oxide/Maghemite Nanocomposite for Detection of Hydrocarbon Vapor Using Surface Plasmon Resonance. IEEE Photonics J..

[126] Fen Y.W., Yunus W.M.M., Talib Z.A., Yusof N.A. (2015). Development of Surface Plasmon Resonance Sensor for Determining Zinc Ion Using Novel Active Nanolayers as Probe. Spectrochim. Acta A.

[127] Matsui J., Akamatsu K., Hara N., Miyoshi D., Nawafune H., Tamaki K., Sugimoto N. (2005). SPR Sensor Chip for Detection of Small Molecules Using Molecularly Imprinted Polymer with Embedded Gold Nanoparticles. Anal. Chem..

[128] Matsui J., Akamatsu K., Nishiguchi S., Miyoshi D., Nawafune H., Tamaki K., Sugimoto N. (2004). Composite of Au Nanoparticles and Molecularly Imprinted Polymer as a Sensing Material. Anal. Chem..

[129] Kumbhat S., Shankaran D.R., Kim S.J., Gobi K.V., Joshi V., Miura N. (2007). Surface Plasmon Resonance Biosensor for Dopamine Using D3 Dopamine Receptor as a Biorecognition Molecule. Biosens. Bioelectron..

[130] Zangeneh Kamali K., Pandikumar A., Sivaraman G., Lim H.N., Wren S.P., Sun T., Huang N.M. (2015). Silver@graphene Oxide Nanocomposite-Based Optical Sensor Platform for Biomolecules. RSC Adv..

[131] Rithesh Raj D., Prasanth S., Vineeshkumar T.V., Sudarsanakumar C. (2016). Surface Plasmon Resonance Based Fiber Optic Dopamine Sensor Using Green Synthesized Silver Nanoparticles. Sens. Actuators B Chem..

[132] Jiang K., Wang Y., Thakur G., Kotsuchibashi Y., Naicker S., Narain R., Thundat T. (2017). Rapid and Highly Sensitive Detection of Dopamine Using Conjugated Oxaborole-Based Polymer and Glycopolymer Systems. ACS Appl. Mater. Interfaces.

[133] Park S.J., Lee S.H., Yang H., Park C.S., Lee C.S., Kwon O.S., Park T.H., Jang J. (2016). Human Dopamine Receptor-Conjugated Multidimensional Conducting Polymer Nanofiber Membrane for Dopamine Detection. ACS Appl. Mater. Interfaces.

[134] Manaf A.A., Ghadiry M., Soltanian R., Ahmad H., Lai C.K. (2017). Picomole Dopamine Detection Using Optical Chips. Plasmonics.

[135] Sharma S., Gupta B.D. (2018). Surface Plasmon Resonance Based Highly Selective Fiber Optic Dopamine Sensor Fabricated Using Molecular Imprinted GNP/SnO_2_ Nanocomposite. J. Light. Technol..

[136] Sun J., Jiang S., Xu J., Li Z., Li C., Jing Y., Zhao X., Pan J., Zhang C. (2019). and Man, B. Sensitive and Selective SPR Sensor Employing Gold-Supported Graphene Composite Film/D-Shaped Fiber for Dopamine Detection. J. Phys. D Appl. Phys..

[137] Yuan Y.J., Xu Z., Chen Y. (2019). Investigation of Dopamine Immobilized on Gold by Surface Plasmon Resonance. AIP Adv..

[138] Wang W., Wang W., Davis J.J., Luo X. (2015). Ultrasensitive and Selective Voltammetric Aptasensor for Dopamine Based on a Conducting Polymer Nanocomposite Doped with Graphene Oxide. Microchim. Acta.

[139] Zhang Z.F., Cui H., Lai C.Z., Liu L.J. (2005). Gold Nanoparticle-Catalyzed Luminol Chemiluminescence and Its Analytical Applications. Anal. Chem..

[140] Li Q., Zheng J.Y., Yan Y., Zhao Y.S., Yao J. (2012). Electrogenerated Chemiluminescence of Metal-Organic Complex Nanowires: Reduced Graphene Oxide Enhancement and Biosensing Application. Adv. Mater..

[141] Liu X., Hu X., Xie Z., Chen P., Sun X., Yan J., Zhou S. (2016). In Situ Bifunctionalized Carbon Dots with Boronic Acid and Amino Groups for Ultrasensitive Dopamine Detection. Anal. Methods.

[142] Liang W., He S., Fang J. (2014). Self-Assembly of J-Aggregate Nanotubes and Their Applications for Sensing Dopamine. Langmuir.

[143] Fang X., Ren H., Zhao H., Li Z. (2017). Ultrasensitive Visual and Colorimetric Determination of Dopamine Based on the Prevention of Etching of Silver Nanoprisms by Chloride. Microchim. Acta.

[144] Tang L., Li S., Han F., Liu L., Xu L., Ma W., Kuang H., Li A., Wang L., Xu C. (2015). SERS-Active Au@Ag Nanorod Dimers for Ultrasensitive Dopamine Detection. Biosens. Bioelectron..

[145] Dong J.X., Li N.B., Luo H.Q. (2013). The Formation of Zirconium Hexacyanoferrate(II) Nanoparticles and Their Application in the Highly Sensitive Determination of Dopamine Based on Enhanced Resonance Rayleigh Scattering. Anal. Methods.

[146] Qin W.W., Wang S.P., Li J., Peng T.H., Xu Y., Wang K., Shi J.Y., Fan C.H., Li D. (2015). Visualizing Dopamine Released from Living Cells Using a Nanoplasmonic Probe. Nanoscale.

[147] Taghdiri M., Mohamadipour-taziyan A. (2012). Application of Sephadex LH-20 for Microdetermination of Dopamine by Solid Phase Spectrophotometry. ISRN Pharm..

[148] Sun B., Wang C. (2012). High-Sensitive Sensor of Dopamine Based on Photoluminescence Quenching of Hierarchical CdS Spherical Aggregates. J. Nanomater..

[149] Zeng Z., Cui B., Wang Y., Sun C., Zhao X., Cui H. (2015). Dual Reaction-Based Multimodal Assay for Dopamine with High Sensitivity and Selectivity Using Functionalized Gold Nanoparticles. ACS Appl. Mater. Interfaces.

[150] Hun X., Wang S., Wang S., Zhao J., Luo X. (2017). A Photoelectrochemical Sensor for Ultrasensitive Dopamine Detection Based on Single-Layer NanoMoS_2_ Modified Gold Electrode. Sens. Actuators B Chem..

[151] Helmerhorst E., Chandler D.J., Nussio M., Mamotte C.D. (2012). Real-Time and Label-Free Bio-Sensing of Molecular Interactions by Surface Plasmon Resonance: A Laboratory Medicine Perspective. Clin. Biochem. Rev..

[152] Li Z.Y., Xia Y. (2010). Metal Nanoparticles with Gain toward Single-Molecule Detection by Surface-Enhanced Raman Scattering. Nano Lett..

